# The analysis of a reference value for baroreflex sensitivity and cardiovascular autonomic neuropathy prevalence in a Chinese population

**DOI:** 10.1186/2047-783X-19-8

**Published:** 2014-02-12

**Authors:** Zi-Hui Tang, Fangfang Zeng, Kuangping Ye, Xiaoling Yu, Linuo Zhou

**Affiliations:** 1 Department of Endocrinology and Metabolism, Fudan University Huashan Hospital, NO. 12 Wulumuqi Mid Road, Building 0#, Jing’an District, Shanghai 200040, China

**Keywords:** Reference value, Baroreflex sensitivity, Cardiovascular autonomic neuropathy

## Abstract

**Background:**

Cardiovascular autonomic neuropathy (CAN) is rapidly growing in all populations worldwide. Baroreflex sensitivity (BRS) is easily applied as a diagnostic test to a large number of individuals in the general population. However, no study has reported the normal reference values of BRS for the CAN diagnostic test in a Chinese population. The aim of this study was to estimate the normative reference value of BRS, and assess CAN prevalence in our cross-sectional dataset.

**Methods:**

We conducted a large-scale, community-based, cross-sectional study in a Chinese population. We performed data analysis on 2,092 subjects. Cardiovascular autonomic function was assessed using spontaneous BRS. A total of 349 healthy subjects were used to perform analysis for the reference value for BRS. The CAN prevalence was calculated in the overall sample, and in patients with diabetes mellitus, patients with hypertension and patients with metabolic syndrome.

**Results:**

In the overall sample, the reference value for total power (TP.brs) was more than 1.96 ms/mmHg. The cut-off points of 1.74 ms/mmHg and 2.53 ms/mmHg were set as high frequency (HF.brs) and low frequency (LF.brs), respectively. CAN diagnostic tests based on the reference value were performed. The estimated CAN prevalence in the overall sample was 20.41% using the BRS test. CAN prevalence was 33.18%, 28.69% and 28.57% in patients with diabetes mellitus, patients with hypertension and patients with metabolic syndrome, respectively.

**Conclusions:**

Our findings provided reference values for BRS. Estimated CAN prevalence was high in this Chinese population, which has become a major public health problem in China.

## Background

Cardiovascular autonomic neuropathy (CAN) is rapidly growing in all populations worldwide [1,2]. The disease is not only a major factor in the cardiovascular complications of diabetes mellitus, but also affects many other majority segments of the general population, such as the elderly, and patients with hypertension and metabolic syndrome [1,3,4]. Cardiovascular autonomic (CA) dysfunction has become a major health concern in China following rapid lifestyle changes. In patients with duabetes mellitus, the prevalence of CA dysfunction was 30-60% [2]. The age at onset of CA dysfunction seems to decrease in patients with diabetes mellitus or/and patients with hypertension. Individuals with previously undiagnosed CAN have an unfavorable cardiovascular risk profile, especially in terms of sudden death, indicating a higher risk of cardiovascular disease [1].

Baroreflex sensitivity (BRS) refers to an index of the arterial baroreceptor functional status that measures changes in heart rate according to blood pressure variation. In clinical research, BRS is used to explore the effects of autonomic nervous system alterations [5]. BRS has the advantage that it quantitatively assesses CA activity, and it yields results that are similar to those yielded by a widely acceptable and traditional CA function test – the Ewing’s test [2,6,7]. CA function testing using BRS is simple, noninvasive and reproducible; therefore, it is easily applied as a diagnostic test to a large number of individuals in the general population [2,7].

However, no study has reported the normal reference values of BRS for a CAN diagnostic test in the Chinese population. CAN diagnostic criteria based on the reference value of BRS may be used to estimate CAN prevalence in our dataset. The aim of this study was to evaluate reference values for BRS, and to estimate CAN prevalence in our cross-sectional dataset.

## Methods and materials

### Study population

This study was a CAN factor survey carried out in a random sample of a middle-aged Chinese population [8]. Participants were recruited from rural and urban communities in Shanghai. Survey participants with undiagnosed CAN, aged 30 to 80 years, were included in this study. A total of 3,012 subjects were invited to a screening visit between 2011 and 2012.

Some subjects were excluded from the study to eliminate potential confounding factors that may have influenced their CA function [8]. The exclusion criteria were as follows: 1) history or findings of arrhythmia, and hyperthyroidism or hypothyroidism; 2) pregnancy or lactation; and 3) serious hepatic or renal dysfunctions. Of the 3,102 subjects, complete baseline data were obtained for 2,092 (67.46%) of the participants.

For reference value analysis, 349 healthy subjects have been collected from the 2,092 overall sample. The inclusion criteria included the following: 1) clinically stable condition with no previous medical history of diabetes mellitus, hypertension, dyslipidemia, coronary artery disease, cerebral stroke, heart failure; 2) fasting plasma glucose <100 mg/dl and 2 h plasma glucose <140 mg/dl after a 75-gram oral glucose tolerance test; 3) normal body mass index between 18.5 and 24.9 kg/m^2^; 4) triglycerides <150 mg/dl and high-density lipoprotein (HDL) cholesterol >40 mg/dl; and 5) systolic blood pressure <140 mmHg and diastolic blood pressure <90 mmHg. The exclusion criteria included the following: use of medications that may affect resting heart rate, such as β-receptor blocker, 1 month before the study.

### Measurements

The subjects were interviewed for the documentation of medical histories and medication, history of smoking habits, and laboratory assessment of cardiovascular disease risk factors. All study subjects underwent a complete baseline clinical characteristics evaluation after an 8-hour fast, which included: 1) history and physical examination; 2) heart rate and blood pressure; 3) fasting plasma glucose and insulin; and 4) fasting plasma lipids. Body mass index was calculated with weight in kilograms divided by the square of height in meters. Fasting plasma glucose was quantified by the glucose oxidase procedure. Serum total cholesterol, HDL cholesterol, triglyceride levels, creatinine, and uric acid were measured by an enzymatic method with a chemical analyzer (Hitachi 7600–020, Tokyo, Japan). Low-density lipoprotein cholesterol levels were calculated using the Friedewald formula. The day-to-day and inter-assay coefficients of variation at the central laboratory in our hospital for all analyses were between 1% and 3%. Metabolic sydrome was diagnosed in individuals who met three or more criteria of the updated National Cholesterol Education Program/Adult Treatment Panel III (World Health Organization Western Pacific Region obesity criteria) [9].

### Diagnostic tests

BRS was measured noninvasively, to investigate potential associations between changes in autonomic regulation and body weight and/or blood pressure, via power spectral analysis. Before CA function assessment, participants should avoid alcohol, smoking and coffee for 24 hours to influence a calm and quiet status. Subjects were studied while awake in the supine position after 20 minutes of rest. Testing times were from 08:00 to 11:00 in the morning. A type-I FuDan Project (FDP)-1 heart rate variability BRS noninvasive detection system (Department of Biomedical Engineering of the Fudan University, Shanghai, China) was used with software version 2.0 (Department of Biomedical Engineering of the Fudan University, Shanghai, China) [5]. Electrocardiography, respiratory signals, and beat-to-beat blood pressure were continually and simultaneously recorded for 15 minutes through an electrosphygmograph transducer (HMX-3C, Department of Biomedical Engineering of the Fudan University, Shanghai, China; placed on the radial artery of the dominant arm), and an instrument respiration sensor. To assess vagus nerve activity in addition to total variability of BRS (TP.brs), we selected the high frequency (HF.brs) to represent vagus nerve activity, and low frequency (LF.brs) to represent baroreceptor reflex [7]. The inter-assay and day-to-day coefficients of variation for the above methods were less than 5%.

### Statistical analysis

The Kolmogorov-Smirnov test was used to determine whether continuous variables followed a normal distribution. Variables that were not normally distributed were log-transformed to approximate normal distribution for analysis. The results are expressed as the mean ± standard deviation or median, unless otherwise stated. The characteristics of the subjects according to relevant groups were assessed using one-way analysis of variance for continuous variables and the *χ*^2^ test for categorical variables. Pearson and Spearman analytical methods were employed for correlation analysis of two variables. All BRS variables were described as percentiles. The quantiles were based on the distribution of BRS values where the 5th, 10th, 25th, 50th, 75th, and 95th percentiles were considered. In this study, *P* < 0.05 is considered to be significant. Data were analyzed using SPSS16.0 (Chicago, IL, USA).

## Results

The baseline characteristics of the 2,092 subjects are listed in Table 1. The overall sample included 905 men and 1,187 women (mean age, 60.78 ± 9.25 years). In the overall sample, the prevalence of hypertension, diabetes mellitus, and metabolic syndrome was 46.65%, 21.33%, and 39.82%, respectively. The demographic parameters, parameters of blood glucose, lipid profile and medical history in the healthy subjects were better than that of the overall sample. There were significant greater value of BRS indices in healthy subjects as compared with the overall sample. The mean age of healthy subjects was younger than that of the overall sample. However, other parameters of demographical, glucose, lipid profile, BRS indices and medical history were similar to the overall sample.

**Table 1:** Baseline characteristics of subject

**Variables**	**Overall total sample**	**Healthy subjects**	***P *** **value**
Demographic information			
N	2,092	349	
Age (years)	60.42 ± 8.68	56.37 ± 8.81	<0.001
Gender (male,%)	705 (33.7%)	73 (20.09%)	<0.001
Body mass index (kg/m^2^)	24.21 ± 3.36	21.58 ± 1.99	<0.001
Waist circumference (cm)	85.07 ± 9.70	77.02 ± 6.86	<0.001
Sysytolic blood pressure (mmHg)	127.62 ± 18.68	114.41 ± 10.94	<0.001
Diastolic blood pressure (mmHg)	79.83 ± 9.69	73.74 ± 6.91	<0.001
Laboratory assays			
Fasting plasma glucose (mmol/L)	5.53 ± 1.81	4.64 ± 0.60	<0.001
Plasma blood glucose (mmol/L)	7.67 ± 3.56	5.29 ± 1.10	<0.001
Serum total cholesterol (mmol/L)	5.32 ± 1.00	5.19 ± 0.97	<0.001
Triglycerides (mmol/L)	1.71 ± 0.98	1.10 ± 0.31	<0.001
HDL (mmol/L)	1.36 ± 0.32	1.54 ± 0.33	<0.001
LDL (mmol/L)	3.19 ± 0.77	3.04 ± 0.77	<0.001
Uric acid (μmol/L)	281.21 ± 83.79	240.91 ± 67.80	<0.001
BRS measurement			
Heart rate (beats/min)	72.42 ± 10.13	68.44 ± 8.69	<0.001
TP.brs (ms/mmHg)	9.38 ± 21.94	11.51 ± 11.16	0.026
HF.brs (ms/mmHg)	9.54 ± 22.80	10.97 ± 12.59	0.162
LF.brs (ms/mmHg)	11.87 ± 14.83	14.96 ± 12.83	0.001
Medical history			
Smoking (yes,%)	306 (14.63%)	28 (8.00%)	<0.001
Hypertension (yes,%)	976 (46.65%)	0 (0%)	<0.001
Diabetes mellitus (yes,%)	446 (21.33%)	0 (0%)	<0.001
Metabolic syndrom (yes,%)	833 (39.82%)	0 (0%)	<0.001

BRS, baroreflex sensitivity; HDL, high-density lipoprotein cholesterol; HF, high frequency; LDL, low density lipoprotein cholesterol; LF, low frequency; TP, total power of variance.

**Figure 1:**
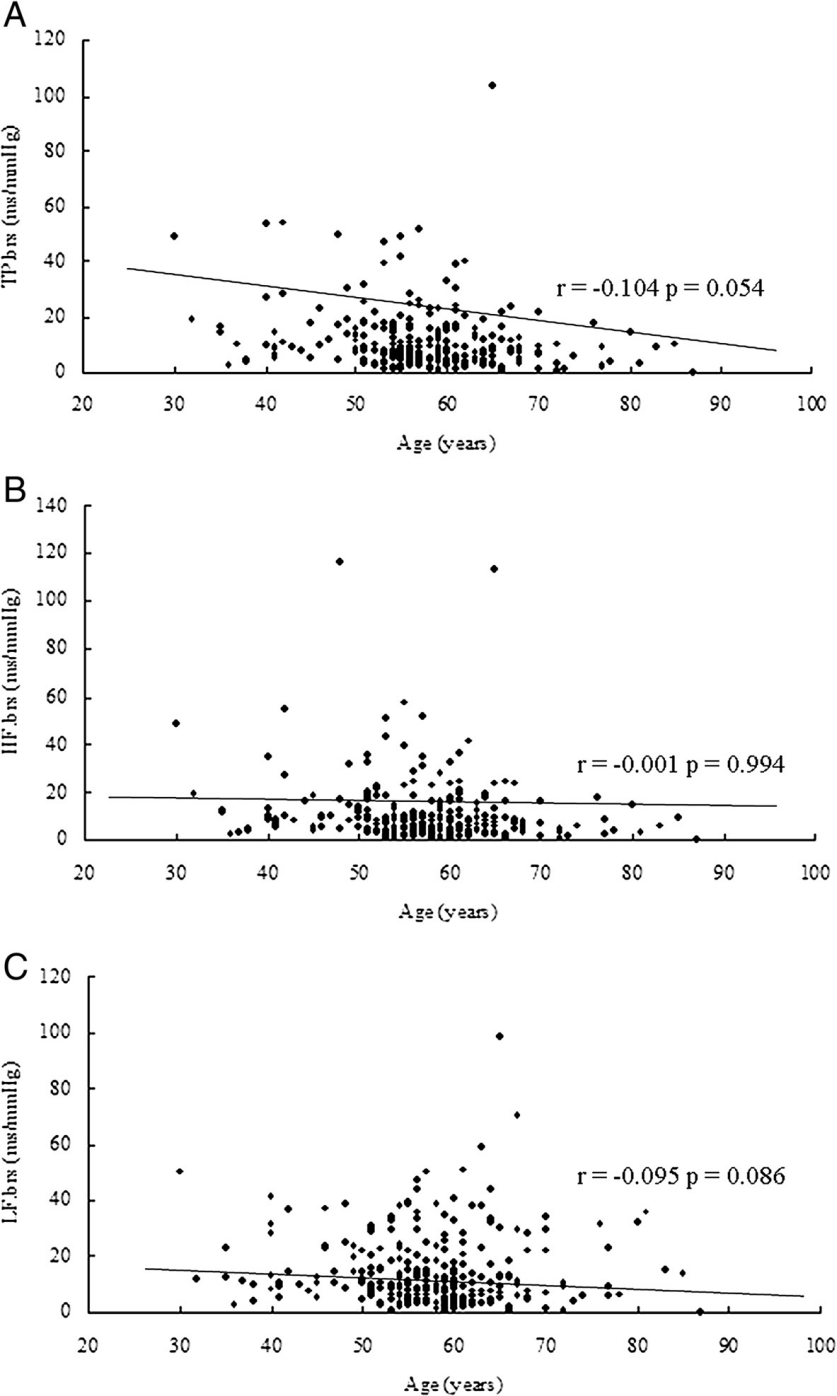
**Results of correlation analysis between age and parameters of baroreflex sensitivity.** Correlation analysis between **(A)** age and total power (TP.brs; r = −0.104, *P* =0.054), **(B)** age and high frequency (HF.brs; r = −0.001, *P* =0.994) and **(C)** age and low frequency (LF.brs; r = −0.095, *P* =0.086).

No normal distribution results were found in BRS indices using Kolmogorov-Smirnov tests (*P* < 0.05 for all, data not shown). In our study, correlation analysis for age and BRS indices showed no significant results for age and TP.brs (r = −0.104, *P* = 0.054; Figure 1A), age and HL.brs (r = −0.001, *P* = 0.994; Figure 1B) as well as age and LF.brs (r = −0.095, *P* = 0.086; Figure 1C). No significant correlations between gender and BRS were found (*P* > 0.05 for all, data not shown).

Reference values of BRS were calculated and are listed in Table 2. In this study, we set the 5th percentile value as cut-off point for TP.brs, HF.brs and LF.brs indices. The reference value for TP.brs was more than 1.96 ms/mmHg. The cut-off points of 1.74 ms/mmHg and 2.53 ms/mmHg were set to HF.brs and LF.brs, respectively. The BRS test was based on reference values of BRS indices to set diagnostic criteria for CAN, which was based on at least one abnormal CA reflex test result.

**Table 2:** Reference values of baroreflex sensitivity

**BRS indices (ms/mmHg)**	**N**	**Mean ± SD**	**5th percentile**	**10th percentile**	**25th percentile**	**50th percentile**	**75th percentile**	**95th percentile**
TP.brs	345	11.51 ± 11.16	1.96	2.66	4.45	8.19	15.54	31.88
HF.brs	346	10.97 ± 12.59	1.74	2.31	3.94	7.38	12.94	33.58
LF.brs	341	14.96 ± 12.83	2.53	3.42	6.08	10.78	21.87	38.58

BRS, baroreflex sensitivity; HF.brs, high frequency; LF.brs, low frequency; TP.brs, total power.

The CAN prevalence in the overall sample was estimated at 20.41% (Table 3). The CAN prevalence was 33.18%, 28.69% and 28.57% in diabetes mellitus patients, hypertensive patients and metabolic syndrome patients, respectively.

**Table 3:** Estimated prevalence of cardiovascular autonomic neuropathy the in different groups

**Group**	CAN*	**Population (N)**	**Prevalence**
Total sample	427	2,092	20.41%
Diabetes mellitus	148	446	33.18%
Hypertension	280	976	28.69%
Metabolic syndrome	238	833	28.57%

*CAN, cardiovascular autonomic neuropathy.

## Discussion

A large-scale, population-based, cross-sectional study was conducted to evaluate reference values for BRS and estimate performance of CAN diagnostic tests among 2,092 participants in the Chinese population. This sample was a good age-adjusted representation of the Chinese population, and the reference values may work similarly well outside the areas studied in China [5,10]. In this study, we provided evidence that the CAN diagnostic test was based on reference values of BRS in a Chinese population.

The results of this study most likely reflect typical BRS patterns for healthy subjects. BRS parameters provide general information on CA function. In this study, reference values and cut-off points of BRS indices were reported. Gerritsen and colleagues [11] conducted a study to analyse reference values for BRS in 191 healthy subjects, and reported that the median TP.brs was 8.8 ms/mmHg and the 10th centile was 4.5 ms/mmHg. Tank and colleagues [12] indicated that the mean ± SD of TP.brs was 8 ± 5 ms/mmHg in 53 subjects without CAN. Another previous study [13] reported that the mean ± SD of this BRS component was 10.4 ± 6.1 ms/mmHg in 52 controls. Our finding are consistent with these previous results. Previous studies [14,15] demonstrated that the mean LF.brs ranged from 11.9 to 14.0 ms/mmHg in a moderate sample. The mean HF.brs was reported to be 18.0 ms/mmHg. In these previous studies, similar variances for the three indices of BRS were reported. Our findings are good representations, and we recommend cut-off points for the normal reference values of the BRS parameters. Autonomic function tests based on BRS and heart rate variability were applied in clinical practice by our research group. Cardiac chemoreflex sensitivity for deactivation of peripheral chemosensors being located in the glomus caroticum is mechanistically related to BRS. Halliwill and colleagues [16] performed a study to assess the activation of peripheral chemoreceptors with acute isocapnic hypoxia which resets arterial baroreflex control of both heart rate and sympathetic vasoconstrictor outflow to higher pressures, resulting in increased heart rate and muscle sympathetic nerve activity without changes in baroreflex sensitivity. Rassaf and collegues [17] performed a study to test whether improvement in renal function following kidney transplantation is related to an improvement in chemosensory function, suggesting that chemosensory activity following kidney transplantation is related to cardiac autonomic control.

Our previous study was conducted to evaluate the performance of the CAN diagnostic test based on a BRS reference value, indicating that these values were contributable to high sensitivity and specificity of the diagnostic test for this disease [18]. In our study sample here, using the BRS test, CAN was estimated at 20.41% in the general population. In patients with diabetes, its prevalence was estimated at 33.18%. The estimated CAN prevalence in patients with diabetes was found to be 20 to 50% in previous studies [2,6], consistent with our finding. In hypertensive individuals, CAN prevalence was estimated at 28.69%. Our previous studies demonstrated that blood pressure and hypertension were strongly associated with low BRS [5]. In this study, estimated CAN prevalence was 28.57% in the metabolic syndrome population. Laitinen and colleagues [19] reported that the prevalence of parasympathetic dysfunction was 25% in subjects with central obesity in subjects with impaired glucose tolerance. Our findings support evidence that CAN has become a serious public problem in China. Higher prevalence of this disease was found in special subgroups.

Several limitations of the study warrant comment. This study did not cover age-groups other than ages 30 to 90 years. In addition, it is important to mention that our study was performed on Chinese individuals, and our findings may not be relevant to people of other ethnicities.

## Conclusions

In conclusion, this study provided reference values for BRS in a Chinese population. Estimated CAN prevalence was high in the general Chinese population, and its prevalence was more frequent in individuals with diabetes mellitus, hypertension, and metabolic syndrome. CAN has become a major public health problem in China.

## Abbreviations

BRS: baroreflex sensitivity; CA: cardiovascular autonomic; CAN: cardiovascular autonomic neuropathy; HDL: high-density lipoprotein; HF.brs: high-frequency baroreflex sensitivity; LF,brs: low-frequency baroreflex sensitivity; TP.brs: total power baroreflex sensitivity.

## Competing interests

The authors declare that they have no competing interests.

## Authors’ contribution

Z-HT designed the study, analyzed data and wrote the manuscript. FZ, KY and LZ contributed reagents, materials and analysis tools. LZ conceived and designed the study. All authors read and approved the final manuscript.
